# Circulating free plasma tumor DNA in patients with advanced gastric cancer receiving systemic chemotherapy

**DOI:** 10.1186/s12907-018-0079-y

**Published:** 2018-11-26

**Authors:** Sávia Raquel Costa Normando, Pamela de Oliveira Delgado, Ana Katherine Soares Barbosa Rodrigues, Waldec Jorge David Filho, Fernando Luiz Affonso Fonseca, Felipe José Silva Melo Cruz, Auro del Giglio

**Affiliations:** 1 Discipline of Hematology and Oncology at ABC Foundation School of Medicine, Santo André, Brazil; 2 Instituto Brasileiro de Controle do Câncer (IBCC), São Paulo, Brazil

**Keywords:** Gastric cancer, Circulating free plasma tumor DNA, Total cell-free circulating DNA, Liquid biopsy

## Abstract

**Background:**

Advanced gastric cancers are usually associated with incurable conditions for which systemic treatments are indicated. Recent studies suggest that circulating cell-free plasma DNA of tumour origin (tDNA) is a promising non-invasive biomarker that can be used to predict the prognosis and monitor the efficacy of systemic treatments in patients with certain types of cancer. We conducted a pilot study to analyse the potential role of tDNA as a biomarker in patients with advanced gastric cancer.

**Methods:**

We included 30 patients with locally advanced unresectable or metastatic gastric cancer. We obtained samples (10 mL of total blood) from each patient every 3 months and performed concomitant CT until disease progression or death. Total cell-free circulating DNA (cfDNA) samples were measured using GeneQuant RNA/DNA Calculator-Amersham Pharmacia Biotech (Biochrom) Ltd. The cfDNA was used to evaluate the ALU DNA sequences 247 and 115. The level of tDNA was calculated from the ratio of the expression of ALU DNA sequences and the concentration of total cell-free DNA. We utilized the RECIST criteria 1.1 to evaluate the tumour response.

**Results:**

Patients with advanced gastric cancer had significantly higher concentrations of cfDNA compared with normal controls (*p* = 0.00015), which allowed us to conclude that the cfDNA in the patients originated from the tumour. We did not find any significant correlation between the level of tDNA and OS or tumour response. However, after the first cycles of chemotherapy (at 3 months), we observed that patients with lower tDNA levels had significantly longer DFS compared with those with higher levels (Cox Regression *p* = 0.0228).

**Conclusions:**

At 3 months after the beginning of chemotherapy, the tDNA levels are correlated with DFS in patients with advanced gastric cancer who receive systemic chemotherapy. tDNA may be a specific, non-invasive and cost effective new biomarker for these patients.

## Background

Gastric cancer (GC) is the fourth most common neoplasm in the world, with an estimated 603,003 new cases diagnosed among men and 330,290 new cases among women each year. However, the majority of GC patients in the United States are symptomatic at diagnosis, and thus, they already present with locally advanced disease that is unresectable or metastatic at the initial manifestation, when there is no possibility for curative treatment. In this context, the overall survival rate of patients with GC at 5 years is approximately 20%, which makes GC the second most common cause of cancer-related death, with an estimated 700,000 deaths per year worldwide [1].

Regarding treatment, surgical resection, which continues to be the primary curative treatment option for GC, results in five-year survival rates of 78 and 34% for stage I and II disease, respectively. Despite this, the overall five-year survival rate for all patients remains low and ranges from 15 to 35% [2].

Because most patients are diagnosed with advanced-stage GC, they require treatment based on palliative chemotherapy, and the goal is to increase quality of life and overall survival. In this context, to monitor the response to treatment, computed tomography (CT) is currently the primary tool used in the diagnosis and clinical management of GC. However, peritoneal dissemination is a more frequent pattern of recurrence or progression in GC and is generally difficult to detect by imaging [3].

Serologic biomarkers such as CA19–9 and carcinoma embryonic antigen (CEA) are currently used to aid in the diagnosis of GC progression or recurrence, as they can be correlated with CT, but these have limitations due to their low sensitivity [4].

Circulating cell-free DNA (cfDNA) resulting from the physiological apoptosis of normal cells can be detected in healthy individuals and is slightly increased in cases of trauma, septicaemia and other diseases such as systemic lupus, pulmonary embolism and myocardial infarction. However, the level of cfDNA in the plasma of cancer patients is two to three times greater than that in the plasma of healthy individuals [5] and includes DNA released into the bloodstream by tumour cells due to various events such as apoptosis, secretion and necrosis.

Huang et al., Frattini et al. and Sozzietal.each monitored cfDNA levels in breast, colon and lung cancers. The results were similar in that they demonstrated that in patients who underwent surgery, the level of cfDNA was decreased significantly after the procedure; therefore, this decrease implies a positive outcome of the postoperative treatment. In contrast, an increase in the levels of cfDNA were correlated with worse symptoms or signs of disease progression, such as the development of metastasis. Therefore, cfDNA could be used as an efficient marker in the control, prediction and evaluation of tumour therapy in such cases [6–8].

Another emerging biomarker that is studied for the monitoring of GC is the fraction of tumour origin of the cfDNA [that is, circulating plasma tumour DNA (tDNA)]. It is known that somatic mutations related to cancer, such as those of the TP53 gene, are specific for malignant neoplasms. It is also known that somatic mutations in GC occur more frequently in the TP53 gene. In this context, the study published by Hamakawa et al. demonstrated that the number of DNA fragments that harbour cancer-specific somatic mutations in the TP53 gene (tDNA) did not correlate with the total level of cfDNA and that only tDNA showed a correlation with disease status in GC patients [9].

Apoptotic cancer cells liberate small DNA fragments that correspond to tDNAin contrast to the total free circulating DNA which is mainly composed of long DNA fragments. Therefore, tDNAmay be estimated at any time point by a ratio of short to long DNA fragments of a particular DNA sequence such as ALU located within the p53 gene DNA.

In this context, we conducted a pilot study to analyse the potential of tDNA to serve as a possible biomarker in patients with metastatic or locally advanced unresectable gastric cancer who undergo chemotherapeutic treatment.

## Methods

Thirty patients with locally advanced unresectable or metastatic gastric cancer that was confirmed by histology and who were treatment-naive or who had been diagnosed with disease progression were included in three Brazilian clinical oncology services–Hospital Estadual Mário Covas (MárioCovas State Hospital), Hospital Anchieta (Anchieta Hospital) and the Instituto Brasileiro de Combate ao Câncer (Brazilian Institute for the Fight Against Cancer)–from May 2014 to January 2016. Peripheral blood samples were prospectively collected from each patient prior to initiation of the established chemotherapy protocol and subsequently every 3 months until disease progression or death; sample collection was concomitant with imaging examinations that assessed the treatment response. Blood samples were drawn through peripheral venipuncture according to the GCLP guidelines. The RECIST 1.1 criteria were used for analysis of the tumour response [10].

Peripheral blood samples from ten normal, age-matched controls were also evaluated for their cfDNA levels to compare with those of gastric cancer patients prior to the start of chemotherapy.

The exclusion criteria were as follows: patients with a) a history of other primary cancers b) severe infection c) trauma in the past 6 months and d) autoimmune diseases.

### Ethics approval and consent to participate

This protocol was approved by our Institutional IRB (number 806.280). All included patients agreed to participate by signing the required informed consent forms.

### Extraction of plasma DNA

A10.0 mL aliquot of whole blood collected in EDTA tubes was centrifuged at 1300 rpm for 10 min. The separated plasma was transferred to a dry tube, which was followed by a second centrifugation at 2400 rpm for 10 min. From this material, 1 mL was collected for extraction. This first centrifugation is necessary to prevent nucleated blood cells from rupturing, while the second is necessary to remove excess proteins from the plasma so that they do not interfere with extraction (DNA purification). The resultant plasma was used for DNA extraction, which was performed using aGFX TM kit (Amersham Pharmacia Biotech, Inc., USA).

In order to minimize genomic DNA, EDTA collected samples were immediately put on special recipients at 4 °C and kept at this temperature until cfDNA extraction. We conducted all DNA extraction steps on the very same day within 4 h of collection.

### Determination of total circulating plasma cell-free DNA (cfDNA) and circulating DNA of tumour origin (tDNA)

The quantitation of cfDNA that was extracted from whole plasma was performed using the *GeneQuant Calculator RNA/DNA-Amersham Pharmacia Biotech (Biochrom)* Ltd., USA.

We ascertained DNA quality by primers devised to amplify the ALU DNA sequences. Primers corresponding to 115 bp amplicons were designed to amplify longer and shorter DNA fragments that would represent the total amount of cell free circulatingDNA (forward:5’CCTGAGGTCAGGAGTTCGAG-3 ‘e reverse 5’CCCGAGTAGCTGGGATTACA-3’). We also designed primers to amplify 247 bp DNA amplicons which represent long DNA fragments liberated from non-apoptotic cells (forward: 5’-GTGGCTCACGCCTGTAATC-3 ‘e reverse 5’CAGGCTGGAGTGCAGTGG-3′). We calculated DNA integrity as the ratio of the concentrations of 247 bp by 115 bp fragments.. The tDNA was calculated according to the ratio between the expression of the ALUs and the total concentrations of cell-free DNA.

### Statistical analysis

The Cox proportional hazards model was used to evaluate the association of the tDNA levels with disease-free survival (DFS) and overall survival (OS). We used the log-rank test for the analysis of the independent groups of tDNA values in relation to OS and progression-free survival (PFS), and we used the Student-T test for evaluation of the mean tDNA levels in patients who responded (partial response + complete response) and in those who did not according to the RECIST criteria. A significance level of 0.05 was adopted. The analyses were performed using the Stata 11.0 statistical package.

## Results

### Characteristics of the population

The studied population had a mean age of 61 years, and most patients were men (66%). Of all the patients, 33% presented with locally advanced unresectable disease at the time of inclusion in the study, and the histology of all tumours was confirmed to be adenocarcinoma. Those with metastatic disease who were included presented with secondary involvement in the following sites: peritoneum, liver and lung.

Seventy-three percent of the patients were included in the study when they received the first line of treatment; patients were included at the time of the start of treatment until the second, third and fourth palliative lines of treatment. The major chemotherapy regimens used were a) FLOX/FOLFOX (30%) b) EOX/ECF/ELF/DCF (20%) c) Carboplatin/Paclitaxel (13%) and d) Paclitaxel monodrug (13%).

Ten patients died before the second collection of the tDNA sample and the second imaging test; the average time from the first collection of tDNA until the death of the total population was 228 days.

Twenty patients were subjected to at least two tDNA collections, of which 40, 30, 15 and 15% provided samples until the second (DNA2), third (DNA3), fourth (DNA4) and fifth (DNA5) samples were collected, respectively.

**Fig. 1:**
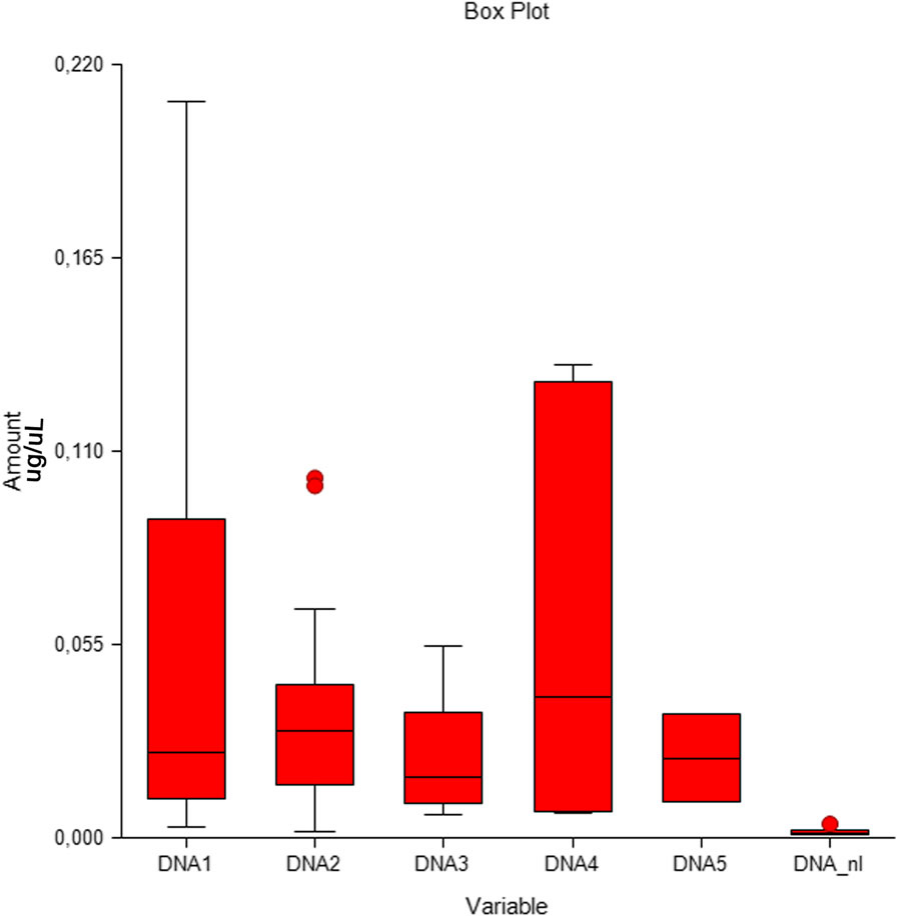
Box plot of the cfDNA distribution in normal controls (DNA_nl) (*p* = 0.00015) and in the study population at baseline (DNA1) and at subsequent quarterly collections (DNA2, DNA3, DNA4 and DNA5) of cfDNA

Ten normal age-matched controls were assessed for gastric cancer and were compared with the gastric cancer patients prior to the start of chemotherapy; significantly lower levels of cfDNA were observed in healthy patients (*p* = 0.00015) (Fig. 1) compared with the patients with GC.

**Fig. 2:**
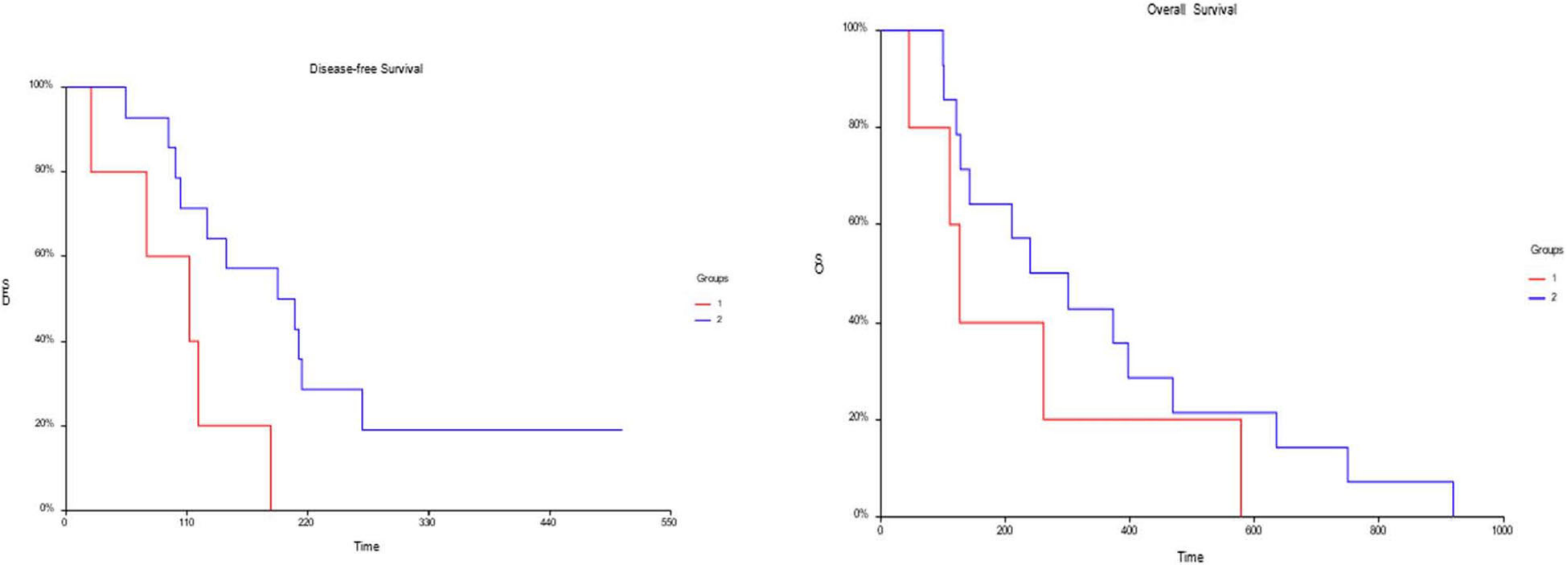
Kaplan-Meier curve for the tDNA collected at the second collection time point and DFS (left curve) and OS (right curve). Group 1 (in red) represents patients with tDNA levels greater than or equal to the 75th percentile, while Group 2 (in blue) represents patients with levels below the 25th percentile. A statistically significant correlation was observed between high levels of tDNA collected at the second collection time point and lower DFS (*p* = 0.0228) but not between high levels of tDNA and OS (*p* = 0.2563)

We did not observe a significant correlation between the tDNA levels in the first (*p* = 0.9892) or second collection (*p* = 0.8132) and tumour response evaluated at the second collection (3 months after the start of chemotherapy). For the Cox model, the tDNA levels collected during the second collection were significantly correlated with DFS (*p* = 0.0220); we also did not observe any significant correlation between the levels of tDNA collected during the first collection and OS or DFS or between the tDNA collected during the second collection and OS. To generate Kaplan-Meier curves for the tDNA levels, disease-free survival (DFS) and overall survival (OS), we arbitrarily separated patients with tDNA levels greater than or equal to the 75th percentile of those with values ​​below the 25th percentile of tDNA values ​​obtained at each collection. We observed that patients with higher levels of tDNA ​​at the second collection had a significantly lower DFS (*p* = 0.0228) (Fig. 2). However, we did not observe a significant correlation between the levels of tDNA and OS (*p* = 0.2563). We also did not observe other significant correlations between the levels ​​of tDNA or cfDNA at other collection time points and DFS or OS.

**Fig. 3:**
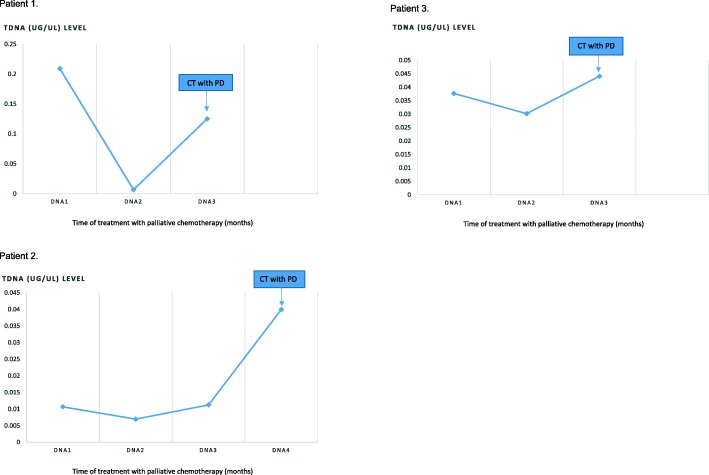
Illustrative level of tDNA (ug/μL) in patient 1, patient 2 and patient 3 over time of treatment, related to progression disease (PD) time according the RECIST criteria 1.1.avaliation utilizing computadorized tomography (CT)

We further illustrated the tDNA levels of three patients (patient 1, patient 2 and patient 3) over time, illustrating the correlation of increased levels of tDNA with disease progression by RECIST1.1 criteria (Fig. 3).

## Discussion

Gastric cancer is often asymptomatic and is correlated with nonspecific symptoms in the early stages; however, by the time symptoms occur, patients usually present with advanced-stage disease [11]. In addition, most of the time, peritoneal metastases are the only signs of disease progression or recurrence, and they are usually not detected early by conventional imaging examinations [12]. Thus, the development of new biomarkers, such as tDNA, which aid in the early detection of gastric cancer progression, can contribute to the clinical management of the disease.

Recent literature has shown that a certain concentration of cfDNA, which is primarily released during apoptosis, may be found in the plasma of healthy individuals. However, it has also been reported that in patients with advanced cancer, tDNA released during necrosis or apoptosis of tumour cells and tumour-adjacent tissues may cause a significant increase in the level of cfDNAin the plasma [13, 14]. These data corroborated our findings, which demonstrated significantly lower cfDNA plasma levels in the control group of healthy patients compared with patients with advanced GC before the start of chemotherapy. Therefore, the cfDNA present in the studied population of GC patients is primarily of tumour origin. To further refine our analysis, we quantitatively established the level of tumour DNA from the analysis of fragment sizes through the relative expression of microsatellite changes that verify DNA integrity. These fragments were classified into small (ALU 115-truncated by apoptosis) and large DNA fragments (ALU247), and after the fraction was calculated, it was applied in relation to the total cfDNA for each sample [15].

Actually, we observed that lower concentrations of tDNA (and not cfDNA) collected during the second collection were significantly related to DFS but not to OS. This finding may indicate greater chemosensitivity and therefore greater tumour eradication in patients with lower levels of tDNA, which may have contributed to a superior DFS. It is therefore possible that tDNA may be useful for monitoring disease recurrence and progression. Additionally, the positive correlation that was observed between DFS and tDNA, but not with cfDNA, clinically corroborates, in a preliminary way, the methodology by which we evaluated the relative amount of tDNA present in each sample.

The absence of a correlation between OS and tumour response could be due to the small number of patients included in this study and to the difficulty in the measurement of tumour response in patients with peritoneal carcinomatosis.

Since this was a pilot study to evaluate the potential usefulness of tDNA for the follow up of advanced Gastric Cancer patients undergoing chemotherapy, we did not attempt to compare our results with common tumour markers such as CEA, CA125 or CA19–9. Therefore, based on our results here presented, we cannot infer if tDNA is or not superior than the other aforementioned common tumour markers.

## Conclusions

We conclude that tDNA values at 3 months after the beginning of chemotherapy correlate with DFS in patients with advanced gastric cancer who receive systemic chemotherapy. tDNA may therefore be a specific, non-invasive and cost- effective new biomarker in these patients.

## Abbreviations

CEA: Carcinoma embryonic antigen
cfDNA: Total cell-free circulating DNA
CT: Computadorized tomography
DFS: Disease free survival
EDTA: Ethylenediaminetetraacetic acid
GC: Gastric cancer
OS: Overall survival
tDNA: Circulating cell-free plasma DNA of tumour origin
